# The Patent Landscape of AI-Based Imaging Diagnostics for Osteoporosis: A Scoping Review

**DOI:** 10.2174/0115734056459449260722064048

**Published:** 2026-07-29

**Authors:** Zhihan Feng, Siruo Wu, Xinyuan Wang, Luxin Lou, Xiaonan Liu, Yuan Chai

**Affiliations:** 1 ORBIT Lab, College of Medicine and Biological Information Engineering, Northeastern University, Liaoning, Shenyang 110016, China; 2 Department of Radiology, Beijing Jishuitan Hospital, Capital Medical University, Beijing, 100035, China; 3 Department of Orthopaedics, Shanghai Sixth People's Hospital Affiliated to Shanghai Jiao Tong University School of Medicine, Shanghai, China; 4 The University of Sydney, Sydney Musculoskeletal Health and The Kolling Institute, Northern Clinical School, Faculty of Medicine and Health and the Northern Sydney Local Health District, Sydney, NSW 2065, Australia

**Keywords:** Osteoporosis detection, Artificial intelligence, X-ray imaging, Deep learning, Patent analysis, Convolutional neural network, Generative adversarial network, Multi-modal data fusion

## Abstract

**Background:**

Osteoporosis is a widespread skeletal disorder characterized by reduced bone density and increased fracture risk. Early detection is critical to preventing disability and healthcare burden.

**Methods:**

A systematic search was conducted in Google Patents for documents filed between June 2022 and June 2025 that applied artificial intelligence or machine learning to osteoporosis detection using X-ray imaging. Patents were screened in three stages by independent reviewers according to predefined inclusion criteria. Eligible patents were analyzed and categorized according to technical objectives and methodological features.

**Results:**

19 patents met the inclusion criteria. Most originated from Asian countries. Deep learning architectures, primarily convolutional and generative models, were central to innovations in bone density estimation, fracture risk prediction, and image preprocessing. Several patents introduced novel approaches such as cross-modal image fusion, anatomical feature transfer, and data augmentation. Persistent challenges included limited interpretability, variability across datasets, and insufficient clinical validation.

**Discussion:**

The identified patents demonstrate growing sophistication in AI/ML-based X-ray analysis, reflecting convergence toward standardized workflows for image enhancement, feature extraction, and risk prediction. Nevertheless, important gaps remain, including limited interpretability, variability across imaging conditions, and insufficient clinical validation. The reliance on complex architectures and synthetic data also raises concerns regarding generalizability and real-world deployment. Addressing these challenges will be essential to ensure reliable, scalable translation of AI/ML-driven osteoporosis diagnostics into routine clinical practice.

**Conclusion:**

The recent patents demonstrate rapid progress in artificial intelligence–driven osteoporosis diagnostics using radiographic imaging. Advances in transparency, model robustness, and clinical validation are essential to enable safe and effective translation of these technologies into clinical practice.

## 1 INTRODUCTION

Osteoporosis is a systemic skeletal disorder characterized by reduced Bone Mineral Density (BMD) and microarchitectural deterioration, leading to elevated fracture risk [1]. It is a prevalent global health issue, affecting approximately 10 million adults over the age of 50 in the United States alone, with another 33.6 million at high risk [2]. Comparable data from the United Kingdom projects that one in two women and one in five men in this age group will sustain a fracture [3]. The condition is primarily associated with hormonal changes, particularly postmenopausal estrogen deficiency, which disrupts normal bone remodeling and accelerates age-related bone loss. Clinically, osteoporosis is often asymptomatic until fragility fractures occur, while radiological findings typically include reduced bone mineral density and deterioration of trabecular bone structure [4].

A fundamental challenge in managing this disease is its insidious progression. Only 25.8% of fragility fracture patients received osteoporosis screening or treatment in the two years prior to fracture, reflecting a major gap in preventive diagnostics [5]. These fractures cause severe pain, disability, and a markedly reduced quality of life, imposing a heavy economic burden on healthcare systems worldwide [6]. This diagnostic delay creates a pressing need for accessible and proactive screening methods that can identify at-risk individuals before the first fracture occurs.

While DXA remains the gold standard for osteoporosis diagnosis [7] by quantitatively measuring BMD to assess bone health, its high cost and limited availability restrict its use for widespread screening, particularly in primary care or rural settings. Consequently, conventional radiography (X-ray) remains a common initial screening and fracture risk assessment tool in clinical practice, despite its relatively low sensitivity to early bone loss. In recent years, advances in Computer-Aided Diagnosis (CAD) have provided new solutions for osteoporosis detection from X-ray images. With rapid developments in deep learning and medical image analysis, Artificial Intelligence (AI) techniques have been applied to osteoporosis diagnosis and shown promise for opportunistic screening [8].

In the context of osteoporosis detection, these applications span multiple aspects of analysis. Convolutional Neural Networks (CNNs) are typically used to automatically extract structural features from radiographic images, such as cortical thickness, trabecular patterns, and bone texture [9, 10]. These features are then used to perform tasks including BMD estimation, osteoporosis classification, and fracture risk prediction [11]. Some approaches also integrate clinical metadata or multi-modal imaging data to improve diagnostic accuracy and model robustness [12]. Deep learning models show promise for opportunistic screening with X-rays, enabling tasks such as automated segmentation of osteoporotic changes [13]; estimation of BMD from standard X-rays, which has been proposed to reduce reliance on DXA [14]; and analysis of trabecular structure [15], all aimed at identifying at-risk patients before fracture.

While several literature reviews have discussed the use of AI in osteoporosis diagnosis [16, 17], an analysis of the patent landscape, which reveals commercial trends and strategic R&D directions, remains limited. Unlike original research articles that primarily report experimental results or algorithmic performance, patents provide insights into technological translation, intellectual property strategies, and emerging directions toward commercialization and clinical deployment. Therefore, examining the patent landscape can complement existing academic literature by highlighting how AI/ML-based osteoporosis detection technologies are being developed and protected for real-world applications. To bridge this gap, this scoping review maps AI and machine learning technologies for osteoporosis detection in X-ray imaging, addressing the following:

(1) What are the main technical tasks targeted by AI-related osteoporosis detection patents using X-ray imaging filed between June 2022 and June 2025?

(2) What obstacles and opportunities for improvement are anticipated for future patent developments in this field?

## 2 METHODS

### 2.1 Patent Searching and Screening

We included patents in this review if they explicitly described the application of artificial intelligence or machine learning to X-ray (radiograph) imaging for osteoporosis diagnosis or screening. Patents were excluded if they: a) did not involve AI/ML-assisted methods; b) did not consider osteoporosis as the primary diagnostic target; or c) did not use X-ray imaging in the data acquisition process.

A comprehensive search was conducted in the Google Patents database in March 2026 using the keyword “AI osteoporosis X-ray”. Google Patents was selected for its integration records from major patent offices, including the United States Patent and Trademark Office (USPTO), the European Patent Office (EPO), and the World Intellectual Property Organization (WIPO), providing a unified interface for international patents. The search was restricted to documents with a priority date between June 1, 2022 and June 1, 2025. The timeframe from June 2022 to June 2025 was selected based on the observed concentration of relevant patents within this period. The following query was used:

(AI osteoporosis X-ray) before:priority:20250601 after:priority:20220601

No limitations regarding patent status (*e.g*., granted or application pending) or language were imposed during the search. Some retrieved patent documents were originally filed in languages other than English (*e.g*., Japanese, Korean), and machine-translated English versions provided by Google Patents were used for analysis. While this approach enabled broader coverage, it may introduce minor inaccuracies in the interpretation of technical details. To mitigate this, patent descriptions were interpreted conservatively, focusing on clearly stated methodological features.

This initial search yielded 350 potentially relevant patent documents (Fig. **1**). Two independent reviewers (SW and XW) performed a three-stage screening. In the first stage, they assessed patent titles against the inclusion criteria. After removal of duplicates and clearly irrelevant results, 80 patents remained for further evaluation. In the second stage, the reviewers examined the abstracts of these patents, resulting in a subset of 21 patents. In the third stage, three authors (SW, XW and ZF) assessed the full text of the remaining patents, leading to the exclusion of 2 additional patents that did not meet the inclusion criteria. Any discrepancies in eligibility were resolved through discussion and consensus with the senior researcher. Ultimately, 19 patents met the eligibility criteria and were included in this scoping review.

### 2.2 Data Extraction and Charting

For each included patent, relevant information (*e.g*., patent number, patent title, inventor, priority date) was manually extracted and tabulated. We organized key data into two summary tables (Table **1** for basic information and Table **2** for technical details) to facilitate charting of results. No formal quality appraisal or risk-of-bias assessment was performed, as our aim was to map technological innovations rather than to evaluate clinical and scientific efficacy.

To synthesize the findings, the included patents were grouped into the following application domains based on their primary objectives: (1) image preprocessing and quality assurance; (2) segmentation and extraction of specific anatomical structures or regions of interest; (3) bone mineral density estimation and fracture risk prediction using image-derived features (*e.g*., grayscale values, texture analysis); and (4) multi-modal analysis that combines imaging data with clinical information.

## 3 RESULTS

The dataset summarized in Tables **1** and **2** reveals several cross-cutting patterns rather than isolated technical descriptions. CNNs represent the dominant architectural framework used in AI/ML-based osteoporosis detection from X-ray images. Across patents, there is a clear convergence toward three primary clinical objectives: BMD estimation, osteoporosis classification, and fracture risk prediction.

In addition, a growing number of inventions incorporate clinical metadata (*e.g*., age, sex, baseline risk factors), reflecting a shift from image-only models toward integrated diagnostic frameworks. These patterns indicate an emerging standardization of AI-driven radiographic workflows, in which preprocessing, feature extraction, and risk prediction are increasingly combined into unified pipelines.

Geographically, most patents originate from Asian countries, highlighting regional concentration of innovation activity. Within the selected time frame (2022-2025), patent filings are distributed across recent years, indicating sustained innovation activity during this period rather than a single peak year. Most filings are by industrial entities, with fewer from academic institutions or hospital-industry collaborations, indicating that AI/ML-based osteoporosis detection is primarily industry-driven, while academic and hospital partners focus on early-stage research.

A simple qualitative assessment of patent quality was also conducted using legal status and forward citation counts. Most patents remain active or granted, suggesting continued technological relevance, while a smaller proportion are pending or expired. Forward citation counts vary considerably across patents, indicating heterogeneous levels of technological influence. These observations provide contextual insight into patent impact, although they are interpreted cautiously due to the limited dataset and variations in patent reporting practices.

### 3.1 Image Preprocessing and Quality Control

Artifacts in X-ray images, caused by metallic implants, noise, motion, or inconsistent exposure, can significantly impair downstream analysis and weaken diagnostic reliability [18]. Several patents integrate deep learning–based preprocessing to address this challenge. A pipeline combines Generative Adversarial Network (GAN)-based image augmentation and enhancement, CNN-based artifact detection, and multi-stage anatomical segmentation [19]. Bone contours are first segmented and verified using an Active Shape Model (ASM), followed by cortical bone extraction *via* a custom II-Net to derive morphology-based osteoporosis indicators. In a separate patent filed in the same year, another collaborative framework integrates CNN-based artifact detection, contrast enhancement, and shape priors to suppress interference while preserving anatomical integrity [20]. A hierarchical approach uses YOLO for bone localization, Local Binary Pattern (LBP) and ResNet for artifact attenuation, and clinical indices (*e.g*., FRAX) for plausibility validation [21].

Normalization is another critical component of preprocessing, as intensity variations from heterogeneous scanners and acquisition protocols can degrade model robustness [22]. A study addresses this by enforcing standardized exposure parameters and consistent anatomical positioning during acquisition [23]. Temporal normalization through strict Standard Operating Procedures (SOPs) is introduced to ensure the comparability of serial radiographs for longitudinal monitoring [24]. Together, these patents emphasize that preprocessing is essential to generate high-quality inputs for reliable AI-based skeletal assessment.

### 3.2 Anatomical Structure Segmentation and Analysis

Patent strategies for anatomical analysis fall into two major categories. The first employs explicit semantic segmentation to isolate bone structures of interest. Examples include U-Net variants for extracting regions near the clavicle-rib interface, where cortical thinning reflects early systemic bone loss [25], and customized hip segmentation networks that separate cortical and medullary regions for BMD-related morphometric analysis [26]. A more geometry-driven approach employs SegNet-based architectures to segment femoral regions and extract clinically relevant anatomical features, such as hip axis length and femoral neck width [27]. These handcrafted and deep features are subsequently integrated and classified using optimization-based algorithms, enabling osteoporosis discrimination based on structural measurements.

The second approach relies on implicit feature extraction without explicit segmentation. End-to-end CNNs, such as EfficientNet-B4, extract discriminative features directly from full radiographs, guided by internal attention mechanisms that enhance focus on clinically relevant vertebral regions [28]. Methods such as Shapley Additive Explanations (SHAP) further improve interpretability by highlighting key image regions contributing to osteoporosis prediction.

A hybrid dual-branch framework integrates full-image and Region-of-Interest (ROI) analyses, in which feature maps from both inputs are fused to generate a combined prediction [29]. Notably, an image quality assessment mechanism is incorporated to dynamically select between the fused output and the global prediction, enhancing robustness when localized regions are unclear. Another strategy employs anatomical region-based multi-model classification, in which the full radiograph is segmented into multiple anatomical regions (*e.g*., clavicle, vertebrae), and separate classification models are trained for each region [30]. The outputs from these region-specific models are then integrated using an ensemble algorithm to improve overall diagnostic accuracy. A region-specific bone density estimation approach localizes key anatomical landmarks, such as the lumbar vertebrae, from chest radiographs [31]. The extracted regions are used to train AI models with DXA-derived BMD values as ground truth, enabling quantitative prediction of BMD and subsequent assessment of bone abnormality risk.

In summary, segmentation-oriented methods support two complementary objectives: explicit segmentation enables quantitative morphometric assessment, while implicit localization enables automated risk prediction. Both leverage CNNs to model skeletal architecture but differ in their clinical focus-structural quantification *versus* diagnostic decision support.

### 3.3 Cross-Modal Image Mapping and Multi-Modality Feature Fusion

Different imaging modalities offer complementary strengths: X-ray radiography captures global skeletal structure with low cost and high accessibility, Computed Tomography (CT) provides volumetric bone architecture, and Magnetic Resonance Imaging (MRI) reveals soft-tissue composition. To mitigate the limitations of single-modality analysis, recent patents utilize cross-modal mapping and feature fusion strategies to combine structural, morphological, and clinical information within unified AI/ML frameworks.

Deep learning-based fusion methods typically fall into two categories: generative models and feature-level fusion using CNNs [32, 33]. GAN-based approaches learn cross-modal transformations to synthesize missing modality data. For example, a GAN is used to generate CT-like 3D reconstructions from 2D hip X-rays, followed by a Hounsfield Unit (HU)-to-BMD conversion to derive volumetric bone density maps from the synthetic data [23]. A related strategy generates “bone probability heatmaps” from CT-aligned training data to infer BMD distributions directly from radiographs [34]. These methods enhance 2D radiographic analysis by approximating 3D bone structure and improving density estimation [35].

In contrast, CNN-based fusion pipelines focus on integrating imaging features with clinical variables to improve diagnostic precision. A two-stage system is proposed in which spinal radiograph features extracted by EfficientNet-B4 are fused with demographic and biochemical markers using a DeepSurv network to predict fracture risk longitudinally [28]. Similarly, CNN-derived features are integrated with survival modeling to estimate time-dependent probabilities of osteoporotic fractures [36]. These strategies demonstrate that combining imaging signatures with clinical risk profiles yields more clinically meaningful predictions than image-only systems.

### 3.4 End-to-End Decision Support

Beyond image analysis, several patents integrate osteoporosis screening with Clinical Decision Support Systems (CDSS) to enable end-to-end diagnostic workflows [37]. A fully automated pipeline linking skeletal localization (YOLO), BMD estimation (CNN), and treatment suggestions generated through embedded CDSS rules is developed [21]. This concept is extended by coupling AI analysis directly with hospital information systems to support personalized therapeutic planning and follow-up recommendations [24].

In comparison, a partially end-to-end system focused on quality control and automated screening rather than treatment guidance is developed [38]. Their framework employs a Deep Belief Network (DBN) for morphological feature extraction and a CNN-based optimization module that dynamically adjusts acquisition parameters to enhance bone visibility. Although it does not provide therapeutic recommendations, it establishes a closed-loop imaging workflow from acquisition to structured reporting, laying a foundation for future CDSS integration.

## 4 DISCUSSION

### 4.1 Summary of Key Innovations

Despite methodological variability, the identified patents reveal a convergent workflow for AI/ML-assisted osteoporosis assessment: radiographs are used as the primary input, image preprocessing (normalization, enhancement, artifact reduction) ensures data quality, CNN-based architectures dominate feature extraction, and clinical endpoints focus on BMD estimation, osteoporosis classification, fracture risk prediction, and decision support. Many systems enhance model performance by incorporating patient metadata (*e.g*., age, sex) or clinical risk factors (*e.g*., FRAX), and most validate outputs against reference standards such as DXA or longitudinal outcomes, reflecting a shift toward clinically oriented AI pipelines.

Notably, three innovative directions emerge across the patents:

1) ***Cross-modal Learning*:** Some methods leverage auxiliary modalities during training. For example, paired CT–X-ray data are used to infer 3D bone density distributions from 2D projections, reducing dependence on high-radiation CT scans [23];

2) ***Cross-region Bone Density Inference*:** Several inventions use skeletal features from one region to infer systemic bone status. Hip and spine BMD are estimated using cortical metrics from chest radiographs [25], while generalizability is enhanced by stratified augmentation and intensity normalization [39];

3) ***Cross-species Transfer Learning*:** Networks are trained using biomechanically tested animal bones, transferring learned structure–strength relationships to human radiographs [40]. This strategy offers a pragmatic solution to the scarcity of human datasets.

### 4.2 Challenges

Despite promising progress, several technical and translational challenges hinder the clinical deployment of AI/ML-based osteoporosis screening systems. First, most CNN- and GAN-driven architectures operate as opaque “black boxes,” offering limited interpretability and few clinically meaningful explanations of their outputs. This lack of transparency undermines physician confidence and limits clinical trust, which are essential for regulatory approval and adoption in medical workflows [41, 42]. Second, although some patents employ GANs to generate synthetic radiographs for data augmentation and training set expansion, such synthetic images may not fully capture the complex heterogeneity of osteoporotic bone structures or implant-related variations. As a result, models trained on these datasets may exhibit reduced reliability and limited external validity when applied to real-world clinical images [19]. Third, practical deployment remains constrained by system complexity: many high-performing models rely on multi-stream fusion or 3D reconstruction, which require specialized hardware and computational resources that are not widely available, particularly in low-resource healthcare settings. Model generalizability also remains a major obstacle, as variations in imaging protocols, X-ray equipment, and patient morphology introduce distributional shifts that degrade performance across hospitals and populations [43, 44]. Finally, broader translational barriers, including data quality, liability attribution, ethical considerations, and emerging regulatory requirements for medical AI, continue to shape the pathway from prototype development to real-world implementation [44, 45]. The increasing heterogeneity and complexity of medical imaging data can pose challenges for AI/ML model development and may affect model performance and robustness.

### 4.3 Future Trends

Future developments in AI/ML-assisted osteoporosis detection are expected to progress from proof-of-concept systems toward clinically deployable and scalable solutions. A major research direction will be multi-modal data fusion, integrating radiographic information with complementary sources such as CT, MRI, electronic health records, and biochemical markers to improve diagnostic robustness and capture both structural and metabolic aspects of bone health. In parallel, fine-grained image analysis will continue to evolve through cortical thickness modeling, trabecular texture analysis, and biomechanically informed feature engineering, enabling closer alignment between model outputs and clinically relevant bone quality metrics. Improving model generalizability will remain a priority, with increasing adoption of multi-center harmonization, domain adaptation, and transfer learning to mitigate scanner- and population-specific bias [46, 47]. From a deployment perspective, edge-cloud collaborative systems within the Internet of Medical Things (IoMT) may enable real-time inference at the point of care while supporting secure cloud-based model updates and distributed learning capabilities. Together, these advancements indicate a shift toward integrated diagnostic ecosystems that combine automation with explainability, personalization, and interoperability across clinical platforms.

Beyond these technical and clinical trends, several broader considerations also emerge. From an intellectual property perspective, these technological developments may also influence future patenting strategies, as organizations increasingly seek to protect integrated AI/ML-based diagnostic frameworks rather than isolated algorithmic components. Within this context, the patent landscape suggests several emerging strategic patterns. The dominance of industry-led filings indicates a strong focus on translational and commercially oriented applications, where intellectual property protection may be used to secure competitive advantage in AI-driven diagnostic tools. In addition, the concentration of patents within a limited set of technical approaches (*e.g*., CNN-based pipelines for BMD estimation and fracture risk prediction) may reflect incremental innovation strategies, where improvements are built upon established frameworks rather than entirely novel paradigms. Notably, the presence of multiple patents addressing similar clinical objectives suggests potential overlaps in technological scope, which may lead to competitive positioning or defensive patenting strategies. However, given the limited dataset and lack of detailed legal and citation network analysis, these observations are interpreted cautiously.

Ethical considerations should be taken into account when interpreting the development of AI-based osteoporosis detection technologies. One concern is the potential for algorithmic bias, as models trained on limited or unbalanced datasets may perform inconsistently across different populations, including variations in age, sex, or ethnicity [48]. In addition, variability in imaging protocols, image quality, and patient demographics introduces additional data complexity, which may affect model generalizability and robustness.

Unequal access to AI-enabled diagnostic tools may also widen existing disparities between healthcare systems, particularly in resource-limited settings. Furthermore, the use of large-scale medical imaging datasets raises important concerns regarding patient privacy, data security, and regulatory compliance [49]. While these aspects are not the primary focus of this review, they remain relevant to the broader context of translating patented technologies into clinical practice.

## 5 LIMITATION

This scoping review should be interpreted in light of certain limitations. First, the analysis is based on patent literature, which typically emphasizes technical novelty over empirical rigor. Many patents describe conceptual pipelines without reporting detailed validation data, limiting insight into real-world clinical performance. Second, while our search captured patents filed between June 2022 and June 2025, the rapidly evolving AI/ML landscape means that some emerging innovations may not yet be reflected in patent databases. Transformer-based architectures are less frequently observed within the analyzed patent set compared with CNN-based approaches. However, this observation is based on a limited sample size and should be interpreted with caution, as it does not necessarily reflect broader technological trends. It may instead be attributed to the relatively recent emergence of transformer models in medical imaging research and the time required for such technologies to translate into patentable clinical applications. Third, because patents do not disclose full implementation details, reproducibility and benchmarking remain challenging. A formal risk-of-bias assessment was not performed. Currently, there is no standardized tool for evaluating bias in patent-based scoping reviews, and the nature of patent documents differs substantially from clinical or experimental studies, which represents a limitation of the present work. Future reviews that integrate both patent and peer-reviewed evidence would provide a more comprehensive understanding of technological maturity and translational readiness.

Formal patentometric or bibliometric analyses (*e.g*., citation networks or temporal/geographic trends) were not performed, and a comprehensive multi-database search with optimized Boolean strategies, including evaluation of search sensitivity and precision, was also beyond the scope of this review. These analyses are constrained by the use of Google Patents, which does not provide fully structured and consistently accessible metadata required for citation-based or large-scale patentometric analysis, and by resource and access limitations associated with multi-database retrieval. Additionally, some patents were originally published in languages other than English, and their interpretation relied on machine-translated versions available in the patent database, which may introduce minor interpretation bias.

A formal Technology Readiness Level (TRL) assessment was not conducted due to the limited availability of information on clinical validation and regulatory status in patent documents. However, a qualitative estimation of technological maturity was performed based on the level of system integration, clinical relevance of the outputs, and the extent to which the described methods approximate real-world diagnostic workflows. Based on these criteria, most patents appear to correspond to early-to-intermediate maturity levels (approximately TRL 3-5), with a smaller subset approaching more application-oriented stages (approximately TRL 5-6). While a formal quantitative comparison of patent quality (*e.g*., claims, citations, or legal status) was not performed, selected indicators were examined to provide general contextual insight. Variations in jurisdictional patent law and reporting practices may affect the comparability of metrics such as claim counts and family size. Therefore, these indicators were interpreted cautiously, and the review primarily focuses on the technological characteristics of the patents.

Although the categorization into preprocessing, segmentation, and predictive modeling may appear straightforward, it reflects a consistent workflow observed across the patent corpus. This recurring structure suggests a degree of technological convergence toward standardized AI/ML-driven radiographic analysis pipelines, offering insight into the maturation of osteoporosis detection systems rather than serving as a purely descriptive framework.

## CONCLUSION

This scoping review analyzed 19 recent patents (2022-2025) concerning AI/ML-assisted osteoporosis detection using X-ray imaging. The reviewed inventions demonstrate a growing sophistication in deep learning–based solutions, encompassing cross-modal feature fusion, automated image quality control, and end-to-end clinical decision support. These innovations collectively reflect the maturation of CNN- and GAN-driven methodologies in advancing diagnostic precision and workflow efficiency. Moving forward, achieving greater interpretability, robust clinical validation, and alignment with evolving regulatory standards will be essential for translating these systems from conceptual frameworks to trustworthy clinical tools. With sustained interdisciplinary collaboration and evidence-based refinement, AI/ML-assisted radiographic screening has the potential to become a cornerstone of future osteoporosis management.

## Figures and Tables

**Fig. (1):**
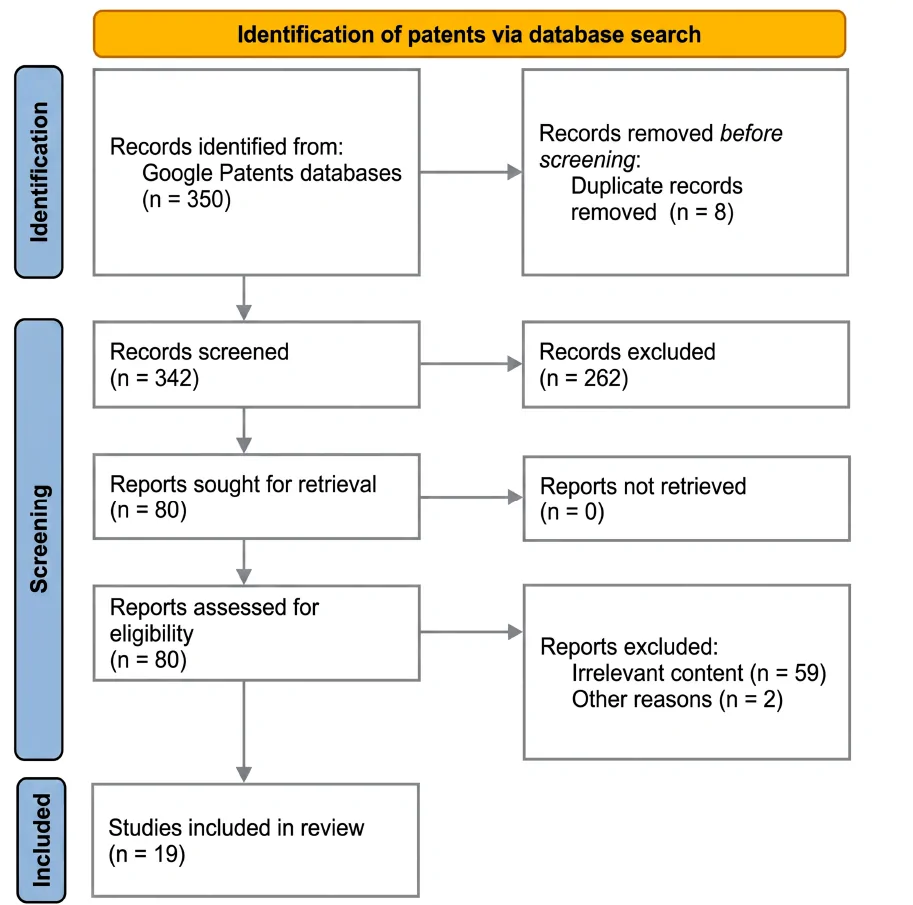
PRISMA flow diagram.

**Table 1:** Patent basic information.

**Inventor(s)**	**Application No.**	**Priority Year**	**Priority Country/ Region**	**Focused Body Parts**	**References**
Tromans *et al*.	PCT/IB2024/056808	2023	*GB	Hip	[19]
Tromans	PCT/IB2024/050708	2023	GB	-	[20]
Lin *et al*.	TW111148208A	2022	*TW	Hip	[21]
Li *et al*.	CN202310376361.5A	2023	*CN	Hip	[23]
Walke	US18/787,498	2023	*US	-	[24]
V.S. *et al*.	PCT/IN2023/051083	2022	*IN	Clavicle	[25]
Yu *et al*.	CN202410094959.XA	2024	CN	Hip	[26]
Paranthaman *et al*.	DE202024105400.8U	2024	*DE	Hip	[27]
Hong *et al*.	KR1020230033364A	2023	*KR	Spine	[28]
Zheng	TW112137279A	2023	TW	Spine	[29]
Zhang *et al*.	CN202580000869.2A	2025	CN	Chest	[30]
Cai	TW112111914A	2023	TW	Spine	[31]
Ke	CN202310400864.1A	2023	CN	Chest, *etc*.	[34]
Kim	KR1020230113033A	2023	KR	Spine	[36]
Zhang	CN202411179277.5A	2024	CN	-	[38]
Honda *et al*.	PCT/JP2024/038382	2023	*JP	-	[39]
Lee *et al*.	KR1020230140986A	2023	KR	-	[40]
Tsai	US18/610,398	2023	TW	Chest	-
Sato	PCT/JP2024/011379	2023	JP	-	-

Abbreviations: * GB = the United Kingdom, TW = Taiwan, CN = China, US = the United States, IN = India, DE = Germany, KR = South Korea, JP = Japan.

**Table 2:** Technical details.

Inventor(s)/Refs.	Machine Learning/Deep Learning Architecture					Imaging/Clinical Input	Output	
	Pre-processing	ROI Detection/ Segmentation	Diagnosis Model	Quantification/ Feature Extraction	Risk Prediction Model		Key Output	Derived Output
V.S. **et al*.* [25]	-	-	-	U-Net, EfficientNet	ML classifiers	X-ray	BMD	Risk assessment
Hong *et al*. [28]	-	-	EfficientNet	-	DeepSurv	X-ray+ baseline	Fracture discrimination score, osteoporosis discrimination score	Risk assessment
Lin *et al*. [21]	-	YOLO	-	ResNet, *LBP	-	X-ray (+baseline)	Density/Texture *SCV	Risk assessment
Zhang *et al*. [30]	CNN	-	-	DBN	-	X-ray	Medical index scoring	Risk assessment
Honda *et al*. [39]	-	-	-	-	-	X-ray	Generated learning image, selected image set	Trained ML model, BMD
Walke [24]	-	-	-	-	-	X-ray	Clinical information	Treatment recommendation
Yu *et al*. [26]	-	Semantic segmentation network	-	-	-	X-ray	Bone thickness	Osteoporosis diagnosis
Tsai	-	-	-	-	-	X-ray	BMD	Risk assessment
Ke	-	CNN (1st)	-	-	CNN (2nd)	X-ray	BMD	-
Tromans [19]	-	-	-	-	-	X-ray/CT/ MRI	Bone parameters (length/width/orientation/ BMD)	Treatment recommendation
Sato	-	-	-	CNN	-	X-ray+ baseline	Bone condition (T-score/BMD/fracture risk)	Bone age, future bone condition
Lee *et al*. [40]	-	-	-	-	-	X-ray	Bone strength	Treatment recommendation
Tromans *et al*. [19]	GAN	(*VGG-) CNN	-	*ASM, II-Net	CNN/Vision Transformer	X-ray	BMD	Bone health prediction
Li. *et al*. [23]	-	-	-	GAN	-	X-ray	3D-BMD	-
Kim *et al*. [36]	-	-	-	CNN	Random Survival Forest/ DeepSurv/ *Cox PH	X-ray	Fracture possibility and timing	Visually marked images
Zheng [29]	-	-	CNN	CNN	-	X-ray	BMD	-
Zhang *et al*. [38]	-	-	CNN	CNN	-	X-ray	Osteoporosis classification	-
Cai [31]	-	-	-	-	-	X-ray	BMD	Risk assessment
Paranthaman *et al*. [27]	*NLM	SegNet	*TJFO	CNN, *GLCM, *LVP	-	X-ray	Osteoporosis classification	-

**Abbreviations:** *LBP=Local Binary Pattern, SCV=Skeletal Characteristic Value, VGG=Visual Geometry Group, ASM=Active Shape Model, II-Net=Iterative Inference Network, Cox PH=Cox Proportional-Hazards, NLM=Non-Local Means, TJFO=Tuna Jellyfish Optimization, GLCM=Gray-Level Co-occurrence Matrix, LVP=Local Visual Pattern.

## Data Availability

All data supporting this review are included within the article.

## LIST OF ABBREVIATIONS

AI: Artificial Intelligence
ASM: Active Shape Model
BMD: Bone Mineral Density
CAD: Computer-Aided Diagnosis
CDSS: Clinical Decision Support System
CNN: Convolutional Neural Network
CT: Computed Tomography
DBN: Deep Belief Network
DR: Digital Radiography
DXA: Dual-energy X-ray Absorptiometry
GAN: Generative Adversarial Network
HU: Hounsfield Unit
II-Net: Iterative Inference Network
IoMT: Internet of Medical Things
LBP: Local Binary Pattern
ML: Machine Learning
MRI: Magnetic Resonance Imaging
PH: Proportional Hazards
ROI: Region of Interest
SCV: Skeletal Characteristic Value
Shapley: Shapley Additive Explanations
SOP: Standard Operating Procedure
VGG: Visual Geometry Group
TRL: Technology Readiness Level

## STANDARDS OF REPORTING

PRISMA guidelines and methodology were followed.

## SUPPLEMENTARY MATERIAL

PRISMA checklist is available as supplementary material on the publisher’s website along with the published article.
